# Updated Genomic Epidemiologic Description of *Candida (Candidozyma) auris*, United States

**DOI:** 10.3201/eid3205.250760

**Published:** 2026-05

**Authors:** Lindsay A. Parnell, Amanda Ribeiro Dos Santos, Kaitlin Forsberg, Meghan Lyman, Elizabeth Misas, Lalitha Gade, D. Joseph Sexton, Anastasia P. Litvintseva, Nancy A. Chow

**Affiliations:** Centers for Disease Control and Prevention, Atlanta, Georgia, USA

**Keywords:** *Candida auris*, *Candidozyma auris*, fungi, antimicrobial resistance, whole-genome sequencing, genomic epidemiology, antifungal resistance, echinocandin resistance, *FKS1*, United States

## Abstract

The multidrug-resistant yeast *Candida* (*Candidozyma*) *auris* has caused several healthcare-associated outbreaks in the United States. We provide a genomic epidemiologic description of 1,535 *C. auris* isolates collected in the United States during 2013–2022. We identified clades I, II, III, and IV but not clades V or VI. Median pairwise single-nucleotide polymorphism distances indicated lower intraclade relatedness for clades I (91), III (43), and IV (43), compared with clade II (1,455). Phylogenetic analysis showed regional clusters with varying predominant clades. Of 809 isolates that underwent antifungal susceptibility testing, 53 were echinocandin resistant, distributed across 3 clades; 92% (49/53) had *FKS1* hotspot mutations, which varied regionally. Our findings corroborate ongoing transmission and clonal expansion of *C. auris*, likely propagated by multiple introductions within and between geographic regions. Echinocandin resistance in multiple clades highlights the need to increase awareness, improve treatment practices, and engage in rapid public health response.

*Candida* *auris,* reclassified as *Candidozyma auris* to reflect its updated phylogenetic position (*1*), is a fungal pathogen characterized by high rates of multidrug resistance and high transmissibility in healthcare settings. More than 40 countries have reported cases; many experienced outbreaks of invasive infection associated with high mortality rates (*2*). The World Health Organization has categorized *C. auris* as a critical-priority fungal pathogen, driven by the need to mitigate spread by scaling up research, scientific development, and public health response efforts (*3*).

Genomic sequencing has been pivotal for understanding global *C. auris* emergence and transmission. Early phylogenetic studies revealed a genetically diverse species that could be classified into 4 major clades hypothesized to have emerged independently and simultaneously from South Asia (clade I), East Asia (clade II), Africa (clade III), and South America (clade IV) (*4*). Clade V was identified later in Iran, and clade VI was reported in Singapore and Bangladesh (*5*–*7*). Some countries have reported the circulation of multiple clades, underscoring the role of travel-related healthcare as an important driver of introductions into new geographic areas (*8*–*11*). An early US study demonstrated the presence of clades I–IV and occurrence of multiple introductions, some of which were linked to seeking healthcare abroad (*8*). Rapid local transmission driven by patient colonization and persistence in the healthcare environment has also been widely reported; low intraclade genetic diversity identified in our study supported those findings (*12*).

*C. auris* clade typing has enabled further characterization of strains of public health and clinical importance, highlighting clade-specific microbiologic and epidemiologic properties. Clades I, III, and IV have primarily been associated with outbreaks, invasive infections, and antifungal resistance; clade II has not (*8*,*13*–*17*). In an early international genomic epidemiologic study, clade I exhibited the highest levels of multidrug resistance to 3 major antifungal drug classes: triazoles (fluconazole), polyenes (amphotericin B), and echinocandins (anidulafungin, caspofungin, and micafungin) (*12*). High fluconazole resistance has consistently been reported in clades I and III (*12*,*16*,*18*,*19*). Clade IV resistance is commonly characterized as variable, both geographically and by drug class (*19*). For example, azole and amphotericin B resistance is reportedly low in the midwestern United States, where clade IV is common (*20*). However, increased resistance has been observed in Colombia among clade IV isolates; resistance patterns varied by region (*21*–*23*). There have been fewer reports of clades II, V, and VI and, among those reported, the strains have generally exhibited low antifungal resistance (*24*–*26*).

Although echinocandin resistance remains low in the United States, increasing pan-resistance and echinocandin resistance remains a concern because echinocandins are the recommended first-line therapy for *C. auris* infection. Researchers hypothesize that patients acquire resistance through antifungal pressure from exposure to echinocandin medications; strains develop substitutions in hotspot regions of *FKS1,* encoding a fungal cell wall protein and an echinocandin target (*27*). Echinocandin resistance has also been documented in drug-naive patients, suggesting the potential for resistant strain transmission (*20*,*28*). Of note, the Clinical and Laboratory Standards Institute gives precedence to *FKS1* hotspot mutations over phenotypic resistance for predicting clinical failure (*29*).

*C. auris* is nationally notifiable in the United States, but reporting of cases varies by jurisdiction. The US Centers for Disease Control and Prevention (CDC) Antimicrobial Resistance Laboratory Network (AR Lab Network), which provides nationwide testing to detect and respond to cases, supporting public health response efforts, conducts complementary laboratory-based testing (*30*). We provide an update on the genomic epidemiology of *C. auris* in the United States, integrating a convenience sample of sequenced cases collected during 2013–2022 with previously reported US sequences. We highlight phylogeographic, phylotemporal, and antifungal resistance patterns observed within each *C. auris* clade.

## Methods

### Cases

AR Lab Network regional laboratories forwarded isolates representing clinical and screening cases from 32 states and jurisdictions to CDC. Each regional laboratory provides antimicrobial resistance testing, including for *C. auris* detection and drug susceptibility. CDC prioritized 1,162 isolates for whole-genome sequencing (WGS) to support public health response efforts. WGS selection criteria included cases with recent healthcare exposures outside of the region; that had unique epidemiology; that were from new facilities or regions; whose isolates exhibited resistant MICs (particularly pan- or echinocandin resistance); that were associated with high-priority outbreaks or donor-derived infection investigations; and that were from areas with known cases where no prior WGS data existed. Some cases were sequenced from high-prevalence areas. We confirmed all unique isolates received for this study were *C. auris* by matrix-assisted laser desorption/ionization time-of-flight mass spectrometry, Sanger sequencing, or both. We used a MALDI Biotyper (Bruker Daltonik, https://www.bruker.com) with the MicrobeNet MALDI database (https://microbenet.cdc.gov). We Sanger sequenced the internal transcribed spacer of the isolates.

### Case Metadata

We extracted case metadata for 1,535 US isolates, using a combination of laboratory submissions and case-based surveillance reporting systems (Table). Specimen collection dates and geographic areas were available for all cases in the study. We included all other epidemiologic data (case type, specimen type, age, and sex) as available from the reporting streams. We classified specimens into 8 categories: colonization screening sites, blood, ear, fluid and drainage, indwelling device, respiratory, urine, and wound. We categorized isolates that could not be classified into those 8 groups as other and isolates for which collection site was unavailable as unknown.

**Table:** Patient demographic data and clinical information for isolates sequenced in study of genomic epidemiologic description of *Candida (Candidozyma) auris*, United States

Characteristic	No. (%) cases							
	All, n = 1,535	Central, n = 9	Mid-Atlantic, n = 188	Midwest, n = 234	Mountain, n = 87	Northeast, n = 560	Southeast, n = 181	West, n = 276
Case type								
Clinical	736 (48)	8 (89)	55 (29)	101 (43)	29 (33)	250 (45)	156 (86)	137 (50)
Screening*	728 (47)	1 (11)	120 (64)	132 (56)	58 (67)	266 (48)	16 (9)	135 (49)
Unknown	71 (5)	0	13 (7)	1 (0.43)	0	44 (8)	9 (5)	4 (1)
Specimen type								
Colonization screening sites	727 (47)	1 (11)	120 (64)	132 (56)	58 (67)	265 (47)	16 (9)	135 (49)
Blood	293 (19)	3 (33)	18 (10)	34 (15)	13 (15)	94 (17)	70 (39)	61 (22)
Ear	9 (1)	1 (11)	0	1 (0.43)	0	5 (1)*	1 (1)	1
Fluid and drainage	20 (1)	0	3 (2)	3 (1)	0	9 (2)	1 (1)	4 (1)
Indwelling device	10 (1)	0	0	4 (2)	0	3 (1)	0	3 (1)
Respiratory	80 (5)	0	4 (2)	8 (3)	5 (6)	28 (5)	24 (13)	11 (4)
Urine	210 (14)	2 (22)	21 (11)	35 (15)	8 (9)	75 (13)	34 (19)	35 (13)
Wound	55 (4)	0	8 (4)	7 (3)	2 (2)	16 (3)	17 (9)	5 (2)
Other	89 (6)	2 (22)	1 (1)	9 (4)	1 (1)	48 (9)	9 (5)	19 (7)
Unknown	42 (3)	0	13 (7)	1 (0.43)	0	17 (3)	9 (5)	2 (1)
Age, y								
<21	10 (1)	0	1 (1)	1 (0.43)	0	2	2 (1)	4 (1)
>21 to <64	629 (41)	5 (56)	97 (52)	127 (54)	46 (53)	183 (33)	70 (39)	101 (37)
>65	793 (52)	2 (22)	78 (41)	83 (35)	41 (47)	316 (56)	104 (57)	169 (61)
Unknown	103 (7)	2 (22)	12 (6)	23 (10)	0	59 (11)	5 (3)	2 (1)
Sex								
F	519 (34)	0	72 (38)	72 (31)	31 (36)	199 (36)	54 (30)	91 (33)
M	687 (45)	7 (78)	87 (46)	100 (43)	42 (48)	255 (46)	63 (35)	133 (48)
Unknown	329 (21)	2 (22)	29 (15)	62 (26)	14 (16)	106 (19)	64 (35)	52 (19)
Year of collection								
2013	1 (0.07)	0	0	0	0	1 (0.18)	0	0
2014	0	0	0	0	0	0	0	0
2015	1 (0.07)	0	0	0	0	1 (0.18)	0	0
2016	100 (7)	0	6 (3)	22 (9)	0	72 (13)	0	0
2017	350 (23)	2 (22)	3 (2)	22 (9)	0	317 (57)	5 (3)	1 (0.36)
2018	123 (8)	1 (11)	3 (2)	54 (23)	1 (1)	59 (11)	4 (2)	1 (0.36)
2019	134 (9)	1 (11)	21 (11)	5 (2)	8 (9)	7 (1)	39 (22)	53 (19)
2020	295 (19)	2 (22)	61 (32)	32 (14)	2 (2)	17 (3)	84 (46)	97 (35)
2021	386 (25)	2 (22)	93 (49)	82 (35)	62 (71)	37 (7)	43 (24)	67 (24)
2022	145 (9)	1 (11)	1 (1)	17 (7)	14 (16)	49 (9)	6 (3)	57 (21)

*One early ear case in the Northeast was counted as a screening case because of circumstances of collection and case definition at the time.

We categorized all US cases as clinical or screening on the basis of available case-based or laboratory-based surveillance data. When that information was unavailable, we used case definitions from the Council of State and Territorial Epidemiologists (*31*). We considered cases that could not be classified as clinical or screening to be unknown.

### DNA Extraction and WGS

We performed DNA extraction and WGS as described previously (*8*), with the following exceptions. We constructed genomic libraries using the NEBNext Ultra DNA Library Prep Kit for Illumina (New England Biolabs, https://www.neb.com) or the DNA Prep Kit (Illumina, https://www.illumina.com), and we sequenced libraries using the HiSeq Rapid SBS Kit v2 (500 cycles), the NovaSeq 6000 SP Reagent Kit, or the MiSeq Reagent Kit v2 (500 cycles) (all Illumina).

### Quality Control, Single-Nucleotide Polymorphism Calling, Phylogenetic Analysis, and Temporal Analysis

We performed quality control, whole-genome single-nucleotide polymorphism (SNP) variant calling, and phylogenetic reconstruction by using the reference-based pipeline MycoSNP-nf version 1.4 (https://github.com/CDCgov/mycosnp-nf). All sequences subjected to variant calling, phylogenetic analysis, and querying for antifungal resistance mechanisms had a minimum mean coverage depth of 20×, a guanine-cytosine content of 42.5%–47%, and a minimum Phred score of 28. For all MycoSNP-nf analyses, we applied downsampling to a coverage of 70 (rate = 0) and used the default haploid parameter. We generated maximum-likelihood phylogenetic trees using the IQ-TREE version 2.1.4 module (https://iqtree.github.io) with Shimodaira-Hasegawa–like approximate likelihood ratio test and ultrafast bootstrap approximation methods.

For the global tree, we aligned 1,162 US sequences and previously reported US (n = 373) and global contextual comparators from Africa, the Americas, Asia, Europe, and Oceania (n = 75) to the clade I reference genome, B11205 (GenBank accession no. GCA_016772135.1), and subjected them to quality control, variant calling, and phylogenetic analysis. For regional trees, we aligned isolates to a clade-specific reference on the basis of the predominant clade in the region. We performed phylogenetic visualizations using Interactive Tree of Life version 7.1 (https://itol.embl.de). We performed temporal clade analyses in R version 4.4.0 (The R Project for Statistical Computing, https://www.r-project.org).

### Intraclade SNP Diversity

We aligned US sequences to their clade-specific references, including B11205 (GenBank accession no. GCA_016772135.1) for sequences identified as clade I, B11220 (GenBank accession no. GCA_003013715.2) for clade II, B11221 (GenBank accession no. GCF_002775015.1) for clade III, and B11243 (GenBank accession no. GCA_003014415.1) for clade IV. We then subjected them to quality control and variant calling using MycoSNP-nf. We used pairwise SNP distance matrices, generated from the SnpDist version 0.8.2 MycoSNP-nf module, for intraclade SNP diversity analysis. To compare intraclade SNP diversity, we calculated the mean SNP distance per isolate within each clade, excluding the reference from comparisons. We visualized distances in clade-specific boxplots using R version 4.4.0.

### Identification of Mutations Associated with Antifungal Resistance

We annotated SNPs obtained with MycoSNP-nf workflow using SnpEff version 5.0 (https://pcingola.github.io/SnpEff) (*32*) and a local SnpEff database for the *C. auris* reference B11205. To identify mutations or polymorphisms only in coding regions, we used the parameters -no-downstream -no-upstream -no-intergenic. We applied filters to the annotated VCF using SnpEffR (https://github.com/CDCgov/snpeffr) to identify mutations and polymorphisms present in the well-known hotspots, including hotspot 1 (F635 to P643), hotspot 2 (D1350 to L1357), and a presumptive hotspot 3 (L686 to N696) as reported previously (*33**,**34*). We annotated regional phylogenetic trees with cases harboring *FKS1* hotspot mutations.

### Antifungal Susceptibility Testing Analysis

The AR Lab Network prioritizes isolates for antifungal susceptibility testing (AFST) as described previously (*20*). In this dataset, AFST was performed on 55% (n = 843/1,535) of the US cases sequenced in this study. The submitting laboratories performed testing using the reference broth microdilution method as described previously (*20*), except for amphotericin B, which was tested by Etest (bioMérieux, https://www.biomerieux.com). We interpreted MICs for fluconazole (n = 836/1,535), amphotericin B (n = 834/1,535), anidulafungin (n = 841/1,535), caspofungin (n = 840/1,535), micafungin (n = 810/1,535), and all echinocandins (n = 809/1,535) as resistant on the basis of CDC-established tentative breakpoints (*35*). We considered echinocandin interpretations not tested if MIC results were unavailable for all 3 echinocandin drugs at the time of the analysis.

### Statistical Analyses

We performed Kruskal-Wallis test with posthoc Dunn test to determine whether pairwise SNP distances differed significantly between clade pairs. We performed χ^2^ tests with standardized Pearson residuals to analyze the associations between clade and various clinical specimen types. We performed all statistical analyses using R version 4.4.0.

### Data Sharing

We submitted reads for newly sequenced cases (n = 1,162), 69 of which were also included in a recently published benchmark dataset (*36*), to the National Center for Biotechnology Information Sequence Read Archive (https://www.ncbi.nlm.nih.gov/sra) under BioProject PRJNA638416 as part of genomic surveillance efforts. Other previously reported US comparators used in this study were deposited under BioProject PRJNA328792 (*4*,*37*), PRJNA796037 (*38*), and PRJNA493622 (*8*). 

This activity was reviewed by CDC and determined by a CDC Human Subjects Advisor to be public health surveillance and not human subjects research. Therefore, institutional review board review was not required.

## Results

### Demographics

We performed WGS for 1,535 *C. auris* isolates from 32 states and jurisdictions within 7 AR Lab Network–defined US regions (Table). We classified 728 (47%) of the sequenced isolates as screening cases and 736 (48%) as clinical cases. Among the clinical cases, common specimen types were blood (293 [40%]), urine (210 [29%]), and respiratory sites (80 [11%]). Among 1,432 cases with reported patient age, the median age was 67 (range 0–99) years. Of 1,206 cases with reported sex, 519 (43%) were female and 687 (57%) were male.

### Phylogenetic and Phylotemporal Characterization

**Figure 1:**
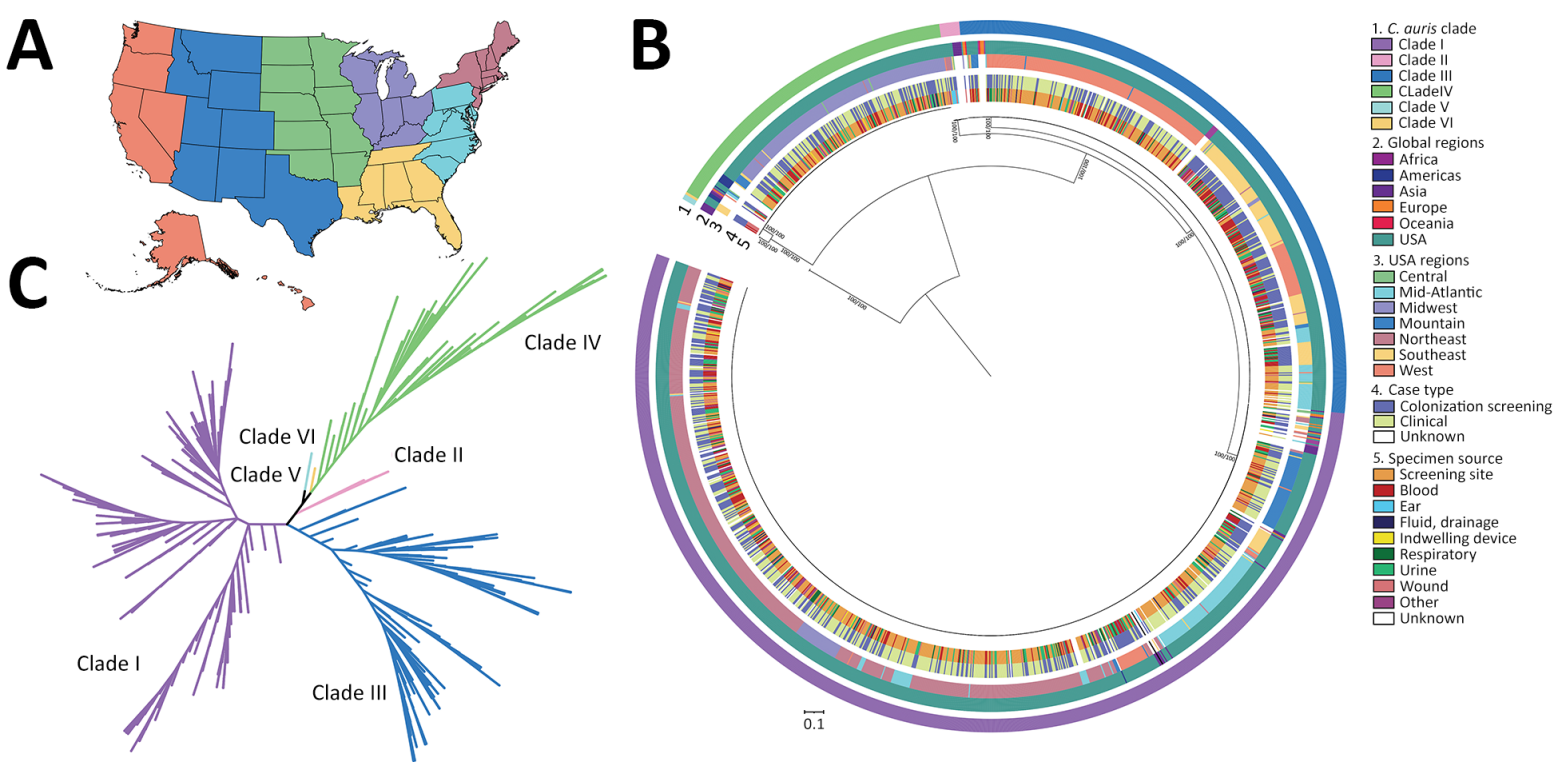
Phylogenetic characterization of sequenced isolates from study of genomic epidemiologic description of *Candida (Candidozyma) auris* in the United States. A) US map showing regions where sequenced cases originated, as defined by the Centers for Disease Control and Prevention Antimicrobial Resistance Laboratory Network (colors defined in section 3 of key at right). B) Maximum-likelihood phylogenetic tree, rooted at the midpoint, represents cases sequenced from the United States (n = 1,535) and various global regions (n = 75). C) Genetic relationships among cases represented as an unrooted phylogenetic tree colored by clade. All US cases cluster within clades I–IV. The phylogenetic trees were inferred from 421,678 whole-genome single nucleotide polymorphisms. Bootstrap support values between major clades were 100, as determined by the IQ-TREE SH-aLRT/UFboot methods.

**Figure 2:**
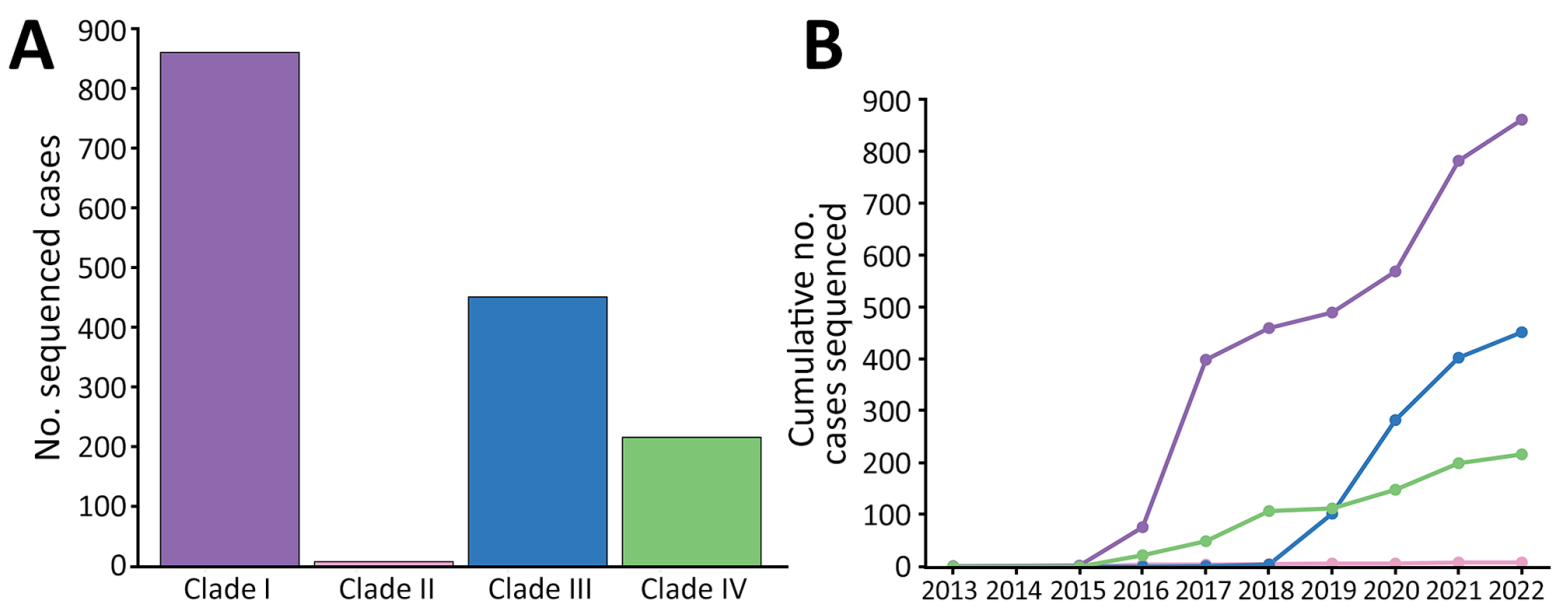
Sequenced isolates by clade and over time from study of genomic epidemiologic description of *Candida (Candidozyma) auris*, United States. A) Isolates by clade: clade I, n = 860; clade II, n = 8; clade III, n = 451; clade IV, n = 216. B) Cumulative number of sequenced US cases of *C. auris* collected from 2013–2022. Colors match clade colors in panel A.

Phylogenetic analysis classified sequenced US cases within clade I (n = 860), II (n = 8), III (n = 451), and IV (n = 216) (Figure 1; Figure 2, panel A). Clade V or VI were not identified (Phylogeny of US and Global Isolates, https://itol.embl.de/tree/15811123643432441764620795). Cases identified from 2013–2016 belonged to clades I, II, and IV. We identified no clade III cases before 2017, then an increase in sequenced clade III cases starting in 2019. By 2017, we identified cases from all 4 clades and >1 clade I, III, and IV case every year thereafter. Clade II appeared infrequently: in 2016 (n = 4), 2018 (n = 1), 2019 (n = 1), and 2021 (n = 2) (Figure 2, panel B).

**Figure 3:**
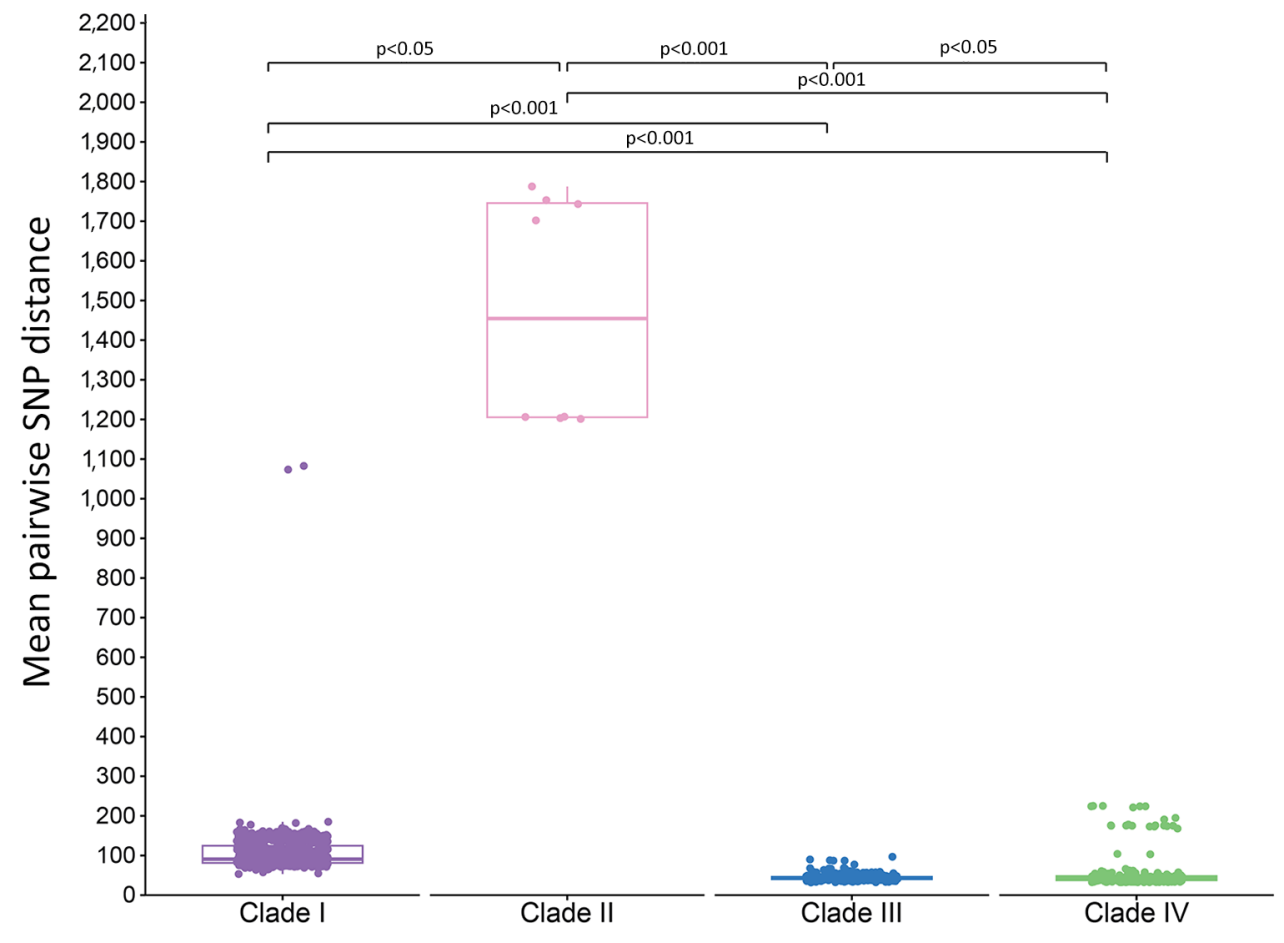
Pairwise SNP distances from study of genomic epidemiologic description of *Candida (Candidozyma) auris* isolates, by clade, United States. Tukey boxplots summarize pairwise SNP distances within each clade; horizontal line within box indicates the median, upper and lower boundaries indicate interquartile range); whiskers indicate largest and smallest distance value within 1.5 times the interquartile range. Dots correspond to each isolate’s mean pairwise SNP distance within its clade. The Kruskal-Wallis test with posthoc Dunn test indicated significant differences in pairwise SNP distances between all clade pairs. Reference strains used were as follows: clade I, B11205 (GenBank accession no. GCA_016772135.1); clade II, B11220 (GenBank accession no. GCA_003013715.2); clade III, B11221 (GenBank accession no. GCF_002775015.1); clade IV, B11243 (GenBank accession no. GCA_003014415.1). SNP, single-nucleotide polymorphism.

We calculated mean pairwise SNP distances for each case within a clade and found distances between all clade pairs differed significantly (Figure 3). Clades III and IV both exhibited a median pairwise SNP distance of 43 (clade III range 32–97; clade IV range 33–225), and clade I had a median pairwise SNP distance of 91 (range 53–1,083). We observed greater SNP diversity in clade II than in any other clade (median 1,455, range 1,201–1,788).

### Resistance

Of 843 cases with AFST, 801 were tested for all 3 antifungal drug classes. Of those, 738 were resistant to >1 antifungal drug. The proportion of resistant cases varied by clade. Clade I and III cases were most frequently resistant; 479/479 isolates of clade I, 1/6 isolates of clade II, 211/212 isolates of clade III, and 47/104 isolates of clade IV were resistant (Appendix Figure 1, panel A). Of the isolates tested for resistance to individual antifungal drugs, 764/836 (91%) were fluconazole-resistant, 97/834 (12%) were amphotericin B–resistant, and 53/809 (7%) were echinocandin-resistant (Appendix Figure 1, panels B–D). For each drug, resistance patterns varied by clade. Of the 809 isolates tested against echinocandins, 49/53 (92%) of echinocandin-resistant isolates had an *FKS1* mutation (Appendix Figure 2), including F635C/Y, S639F/P/Y, and D642Y within hotspot 1; R1354S within hotspot 2; and M690I and W691L within a presumptive hotspot 3. *FKS1* hotspot mutations in echinocandin-susceptible strains were infrequent. We detected mutations in 13/756 (2%) of such cases: in hotspot 1, L638F, S639Y, D642Y; in hotspot 2, L1357F; in hotspot 3, D687V and M690I. In addition, 8 cases without reported AFST harbored an *FKS1* hotspot mutation, including L638F, S639P/Y, L1357F, and D642Y.

### Regional Analysis

**Figure 4:**
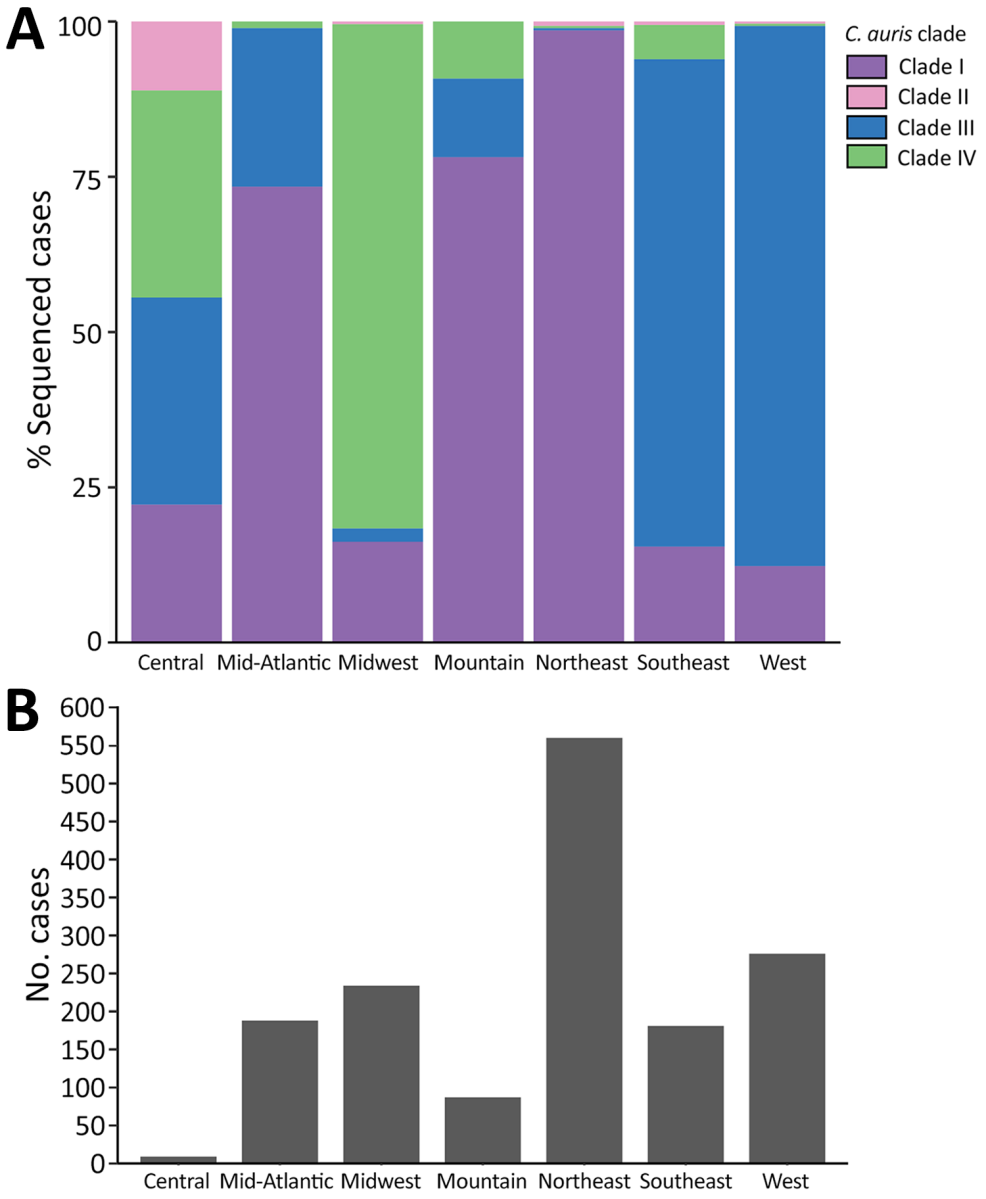
Clades by geographic region from study of genomic epidemiologic description of *Candida (Candidozyma) auris*, United States. A) Percentage of isolates belonging to each clade for each region. B) Total number of cases sequenced in each region.

**Figure 5:**
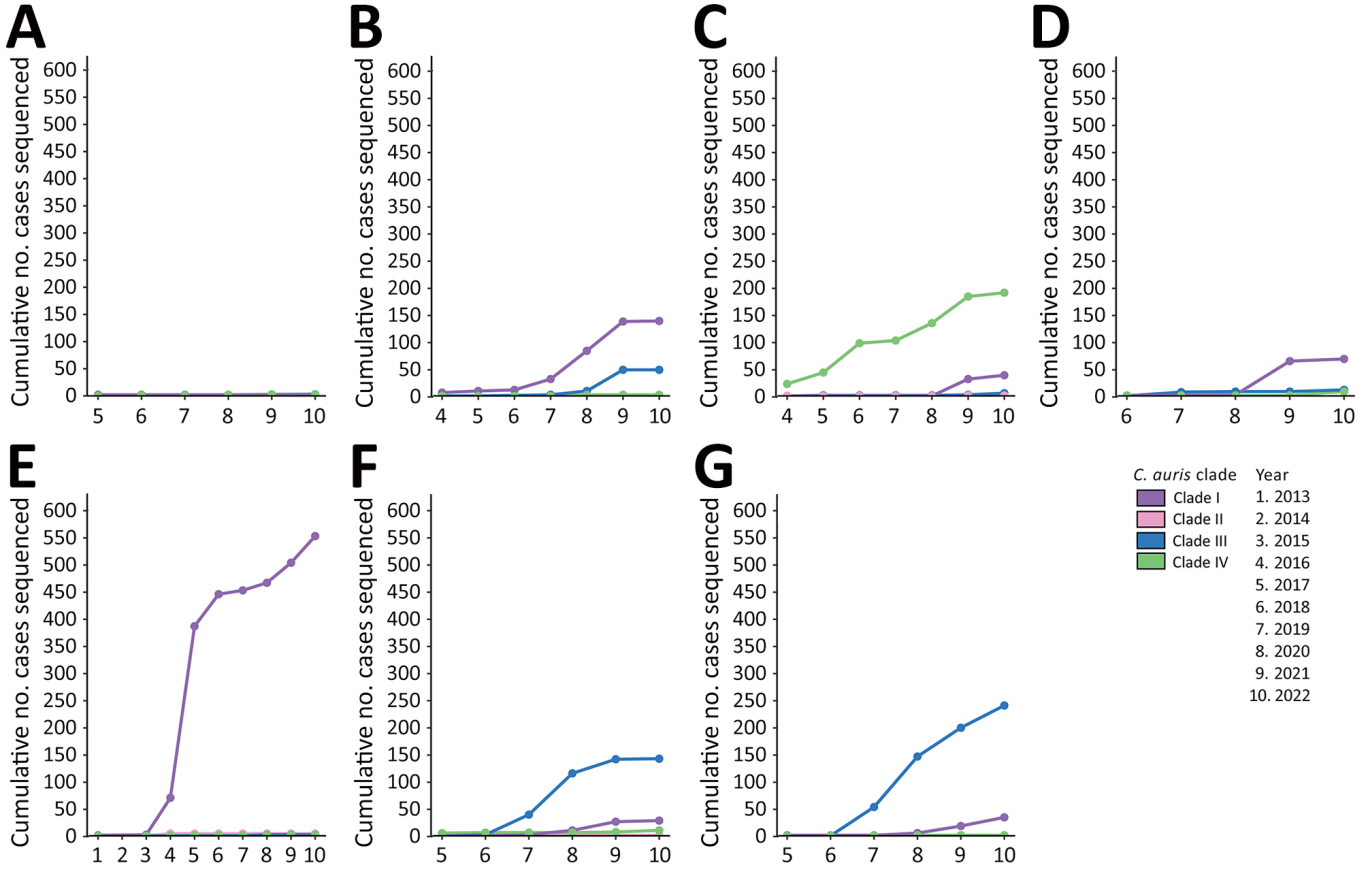
Cumulative number of isolates sequenced per clade, by year and by geographic region, from study of genomic epidemiologic description of *Candida (Candidozyma) auris*, United States. (A) Central; (B) Mid-Atlantic; (C) Midwest; (D) Mountain; (E) Northeast; (F) Southeast; (G) West.

**Figure 6:**
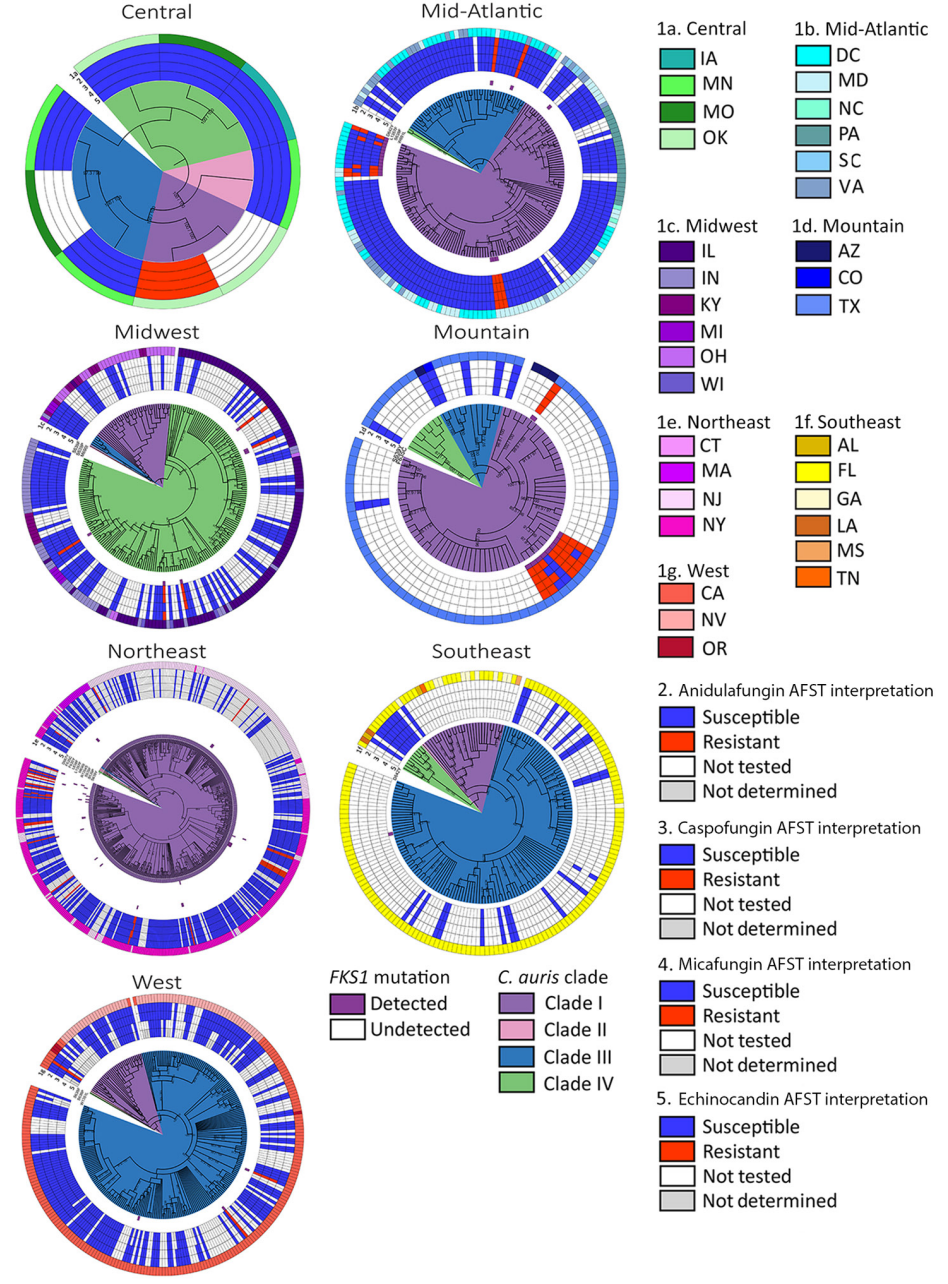
Midpoint-rooted maximum-likelihood phylogenetic trees of sequenced isolates, by region, from study of genomic epidemiologic description of *Candida (Candidozyma) auris*, United States, using clade-specific references. Clades are distinguished on the phylogenetic trees. Branch lengths are annotated with bootstrap support values (Shimodaira-Hasegawa–like approximate likelihood ratio test//ultrafast bootstrap approximation), where Shimodaira-Hasegawa–like approximate likelihood ratio test is >80 and ultrafast bootstrap approximation is >95. AFST, antifungal susceptibility testing.

We observed regional geographic clustering in all clades (Figure 1). Although all regions had multiple clades, most regions had a predominant clade (Figure 4). We identified clade I primarily in the Mid-Atlantic, Mountain, and Northeast regions and clade III primarily in the Southeast and West regions. The Midwest was the only region where clade IV was primarily identified. We sequenced fewer isolates from the Central region (n = 9), and no clade predominated. Within each region, the number of clades sequenced varied over time (Figure 5). We generated phylogenetic trees displaying results by state, clade, and drug resistance findings (Figure 6).

**Figure 7:**
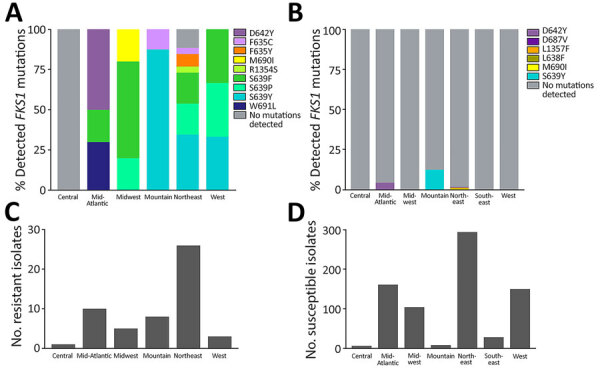
Echinocandin susceptibility by region for isolates from study of genomic epidemiologic description of *Candida (Candidozyma) auris*, United States. A, B) Percentages of isolates with detected *FKS1* mutations among resistant (A) and susceptible (B) isolates. C, D) Total numbers of resistant (C; n = 53) and susceptible (D; n = 756) isolates detected.

We detected an *FKS1* mutation in 100% of echinocandin-resistant cases sequenced in 4 regions: in the Mid-Atlantic, D642Y, S639F, and W691L; in the Midwest, M690I, S639F, and S639P; in the Mountain, F635C and S639Y; and in the West, S639F, S639P, and S639Y (Figure 7). We detected an *FKS1* mutation in 23/26 (88%) of Northeast echinocandin-resistant cases sequenced (F635C, F635Y, R1354S, S639F, S639P, and S639Y). We detected no *FKS1* mutation in the 1 echinocandin-resistant case in the Central region. Detected *FKS1* genotypes varied regionally; the Northeast region exhibited more unique mutations than did other regions.

## Discussion

We present an updated genomic epidemiologic picture of *C. auris* cases from 7 US regions. Consistent with previous genomic epidemiologic survey reporting (*8*), sequenced cases revealed the presence of 4 major clades (I, II, III, and IV). Clades V and VI were absent within the timeframe; clade V has not been reported outside of Iran, and clade VI is a recently reported clade that has been identified in Bangladesh and Singapore (*6*,*7*). However, a likely introduction to Singapore from Bangladesh suggests potential transmission to new areas through travel-related healthcare.

Clades I, III, and IV exhibited relatively low intraclade diversity; median pairwise SNP distances were <100. Even with that level of diversity within the clades, we observed clustering patterns that correlated with the regions and states of collection. That finding suggests that the main transmission patterns of *C. auris* lie within and between healthcare facilities in similar geographic areas. Although most cases observed are likely a consequence of clonal spread, maximum pairwise distances reached 1,083 SNPs for clade I, 97 SNPs for clade III, and 225 SNPs for clade IV, suggesting more divergent strains in these areas. For example, 2 clade I cases identified in the Central region in 2017 were genetically distinct from all other clade I cases sequenced; their mean pairwise SNP distances were 1,073 and 1,083. We did not sequence genetically similar strains to those 2 in this study. It is possible that the strains were not transmitted after their initial introductions or related strains remained undetected, based on our testing strategy.

Clade II exhibited greater intraclade diversity compared with others, despite its early initial introduction into the United States. Temporal analysis revealed sporadic identification of clade II cases throughout the years. Of >700 clinical cases sequenced, 6 were of clade II and demonstrated a strong association with the ear (Appendix Figure 3). That observation is consistent with a study demonstrating a propensity for this clade in ear specimens among 61 cases collected over 20 years (*25*). We saw no evidence of transmission of clade II in our study; that underrepresentation might be rooted in clinical testing practices that limit fungal detection in the ear and other nonsterile sites, unique genetic or biochemical features that make it less virulent and transmissible, or a combination of those factors (*39*). Furthermore, we sequenced cases on the basis of predetermined priorities, which might have limited our ability to capture clade II cases.

We also observed phylogeographic patterns of specific strains found in different states or regions. We identified >2 clades in all regions, but 1 specific clade was more frequently sequenced in most regions. Testing priorities and targeted sequencing strategy likely affect those observed patterns. Nonetheless, the pattern suggests that a clade initially established in an area is sustained through ongoing transmission and is not displaced by other clades. Whether competition between clades plays a role in those observations is unknown.

In some instances, we observed relatedness among strains collected in different regions and states (Figures 1, 6). Interregional and interstate clustering likely stemmed from patient movement or transfers, in which patients sought and received healthcare in other areas. Those data reinforce the role of both local spread and travel-related introductions as important drivers of *C. auris* geographic expansion. Further genomic characterization of phylogeographic patterns is needed to resolve local strain differences and understand how they reflect transmission.

Previous studies have demonstrated the circulation of multiple clades, even within the same facility and patient (*40*); that study described the coexistence of clade I and III in 5 patients in southern Nevada, an area that has experienced large clade I and III outbreaks. However, the clinical implications of multiple clades within a single person are not known. It has been hypothesized that coexisting clades within a single host or geographic area, even those of opposite mating types, might provide conditions for mating and genetic exchanges that lead to more virulent clades and strains. Evidence of recombination signatures among clades is lacking; rather, the high intraclade relatedness observed in our study and population genomic studies suggests clonal expansion (*41*).

We observed drug resistance frequently among clade I and III cases, largely driven by fluconazole resistance (Appendix Figure 1). That finding is consistent with reported susceptibility profiles in areas experiencing clade I and III transmission (*11*,*16*,*42*). We observed echinocandin resistance in multiple clades. Regions where clade I predominated (Mid-Atlantic, Mountain, and Northeast) collectively had 44/53 (83%) of such strains.

As expected, *FKS1* mutations differed across regions (Figure 7). Most likely, those mutations arose independently as a result of antifungal pressure following treatment. However, the mutations do not preclude the possibility of echinocandin-resistant transmission, particularly in highly related strains with common *FKS1* mutations. We could not distinguish between those possibilities because treatment history was unavailable. In addition, some clade I cases in the Central and Northeast regions exhibited echinocandin resistance without an identified *FKS1* mutation, which could warrant further investigation for potential novel non-*FKS1* mutations driving resistance.

Our dataset does not represent overall reported US *C. auris* cases. Although the AR Lab Network tests a substantial volume of cases nationally, some are tested independently by commercial, clinical, and public health laboratories. Therefore, this dataset reflects isolates available through the network based on established testing priorities, which were driven by epidemiologic need and logistical factors. Sequencing of the available isolates focused primarily on cases from new and emerging areas and other topics of interest and was intended to inform public health response. For example, we targeted pan- and echinocandin-resistant isolates, which may result in overrepresentation of such strains in the dataset. Although most isolates in this study represent unique cases, there were exceptions. For example, we sequenced isolates from the same patient on a few occasions to confirm the development of echinocandin resistance following treatment. Overall, because of circumstances surrounding priorities for isolate testing and data availability, phylogeographic and clade-related resistance patterns we described here are not generalizable to the entire *C. auris* population in the United States.

Approximately 55% of isolates in this dataset had accompanying AFST results. Because data availability varies regionally based on the laboratory’s testing strategy and resources, AFST results may be overrepresented or underrepresented in certain jurisdictions. Certain AFST patterns might also be overrepresented in areas experiencing high transmission. For example, *C. auris* strains sequenced in areas in the Midwest, which had frequent clade IV transmission (*43*), likely exhibit similar AFST patterns. Therefore, the resistance frequencies we reported are not intended to be representative of the AFST patterns across the United States.

In conclusion, our data provide a national and regional perspective of *C. auris* clades in the United States and their resistance profiles. A multilevel approach to monitoring clades and echinocandin resistance will inform the targeting of public health resources to mitigate transmission and treat infections.

Appendix Additional information about genomic epidemiologic description of *Candida (Candidozyma) auris*, United States.
