# Supplementary material for: Updated Genomic Epidemiologic Description of Candida (Candidozyma) auris, United States
Source: Emerg Infect Dis. 2026 May;32(5):728–39. doi: 10.3201/eid3205.250760 (PMC13174969; doi:10.3201/eid3205.250760)
Supplement: Appendix — Additional information about genomic epidemiologic description of Candida (Candidozyma) auris, United States. [file 25-0760-Techapp-s1.pdf]

Article DOI: https://doi.org/10.3201/eid3205.250760

EID cannot ensure accessibility for supplementary materials supplied by authors. Readers who have difficulty accessing supplementary content should contact the authors for assistance.

Updated Genomic Epidemiologic Description of *Candida (Candidozyma) auris*, United States

Appendix

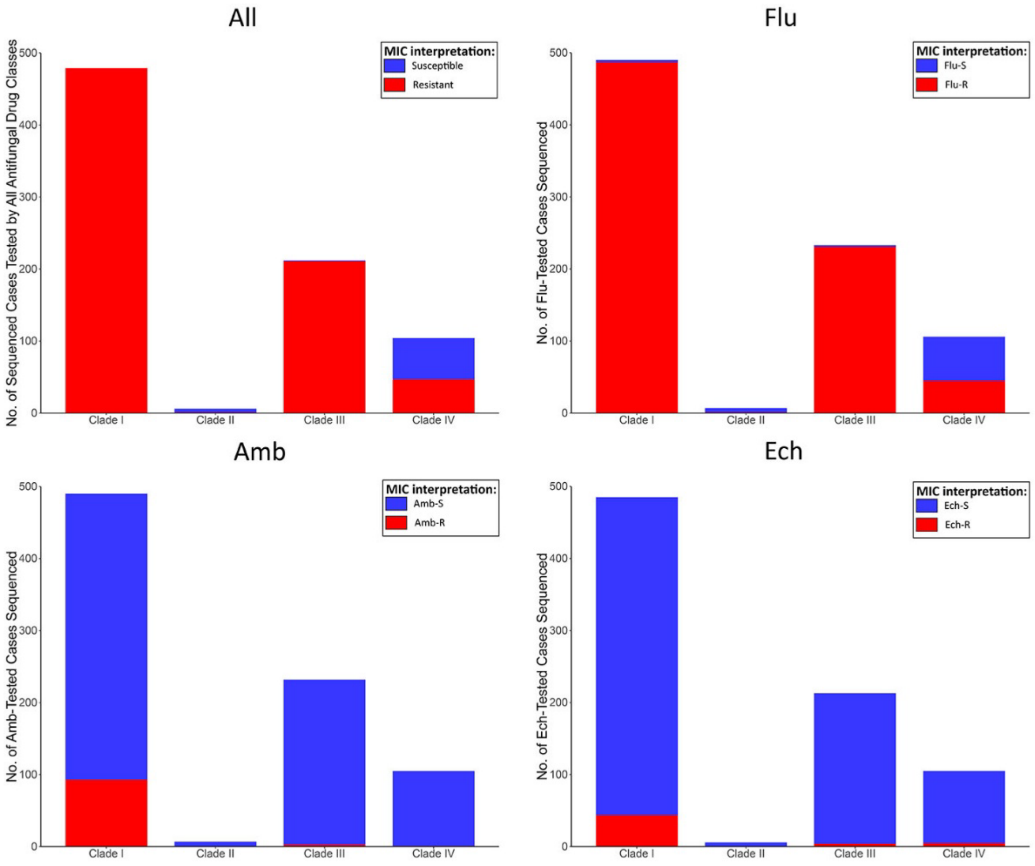

Appendix Figure 1. A) Of 801 cases tested by three antifungal drug classes, the proportion of cases resistant to at least one antifungal drug in Clade I (n = 479/479), Clade II (n = 1/6), Clade III (n = 211/212), and Clade IV (n = 47/104). B) Proportion of flu-s and flu-r cases in Clade I (n = 487/490), II (n = 1/7), III (n = 231/233) and IV (n = 45/106). C) Proportion of amb-s and amb-r in Clade I (n = 93/490), II (n = 0/7), III (n = 3/232), and IV (n = 1/105). D) Proportion of ech-s and ech-r cases in in Clade I (n = 44/485), II (n = 0/6), III (n = 4/213), and IV (n = 5/105). Flu = Fluconazole, Amb = Amphotericin B, Ech = Echinocandin

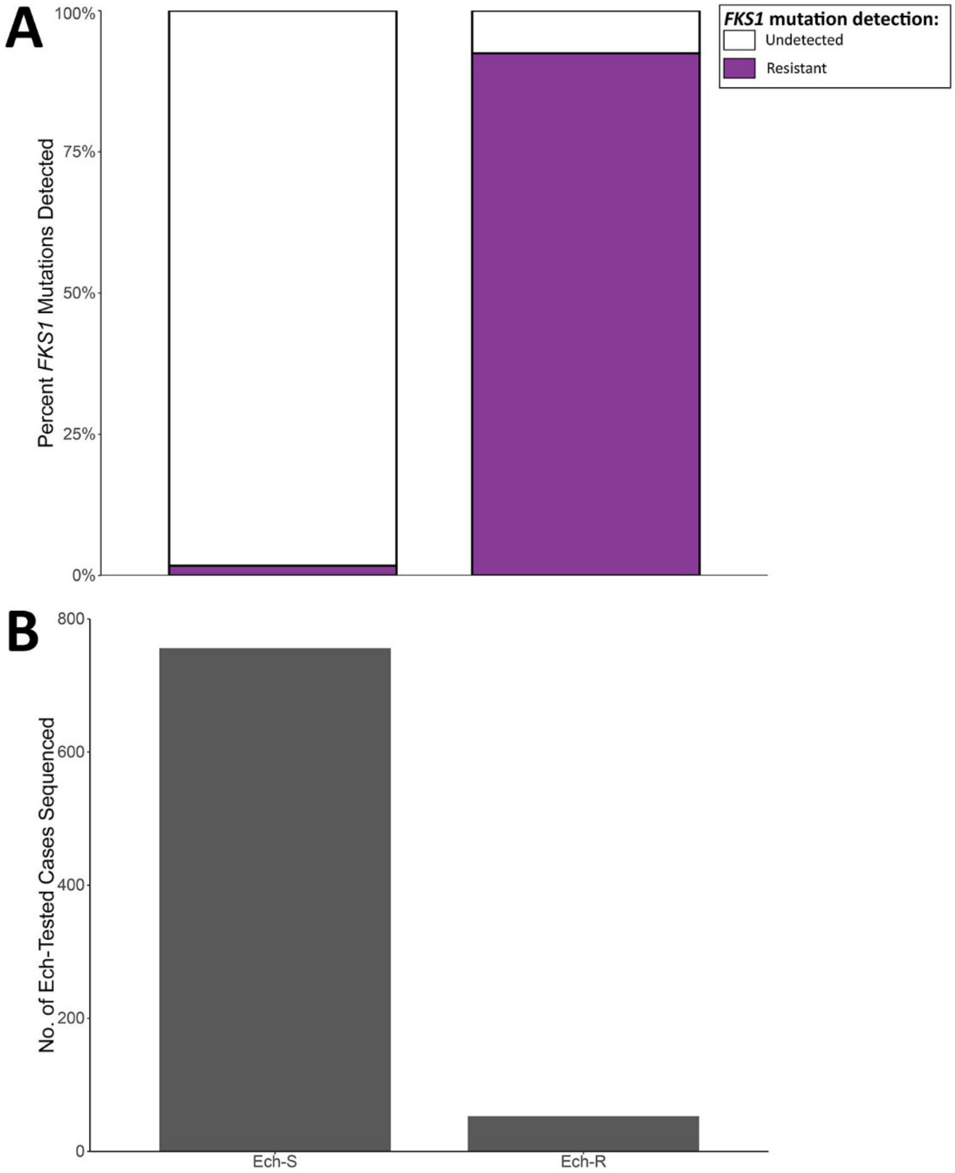

Appendix Figure 2. Percent of total ech-s and ech-r cases with and without a detected *FKS1* mutation. An *FKS1* mutation was undetected in 98% (n = 743/756) of ech-s cases. 92% (n = 49/53) of ech-r isolates harbored an *FKS1* mutation (including S639F/P/Y, F635C/Y, D642Y, R1354S, M690I, and W691L). Ech-R = Echinocandin-Resistant, Ech-S = Echinocandin-Susceptible

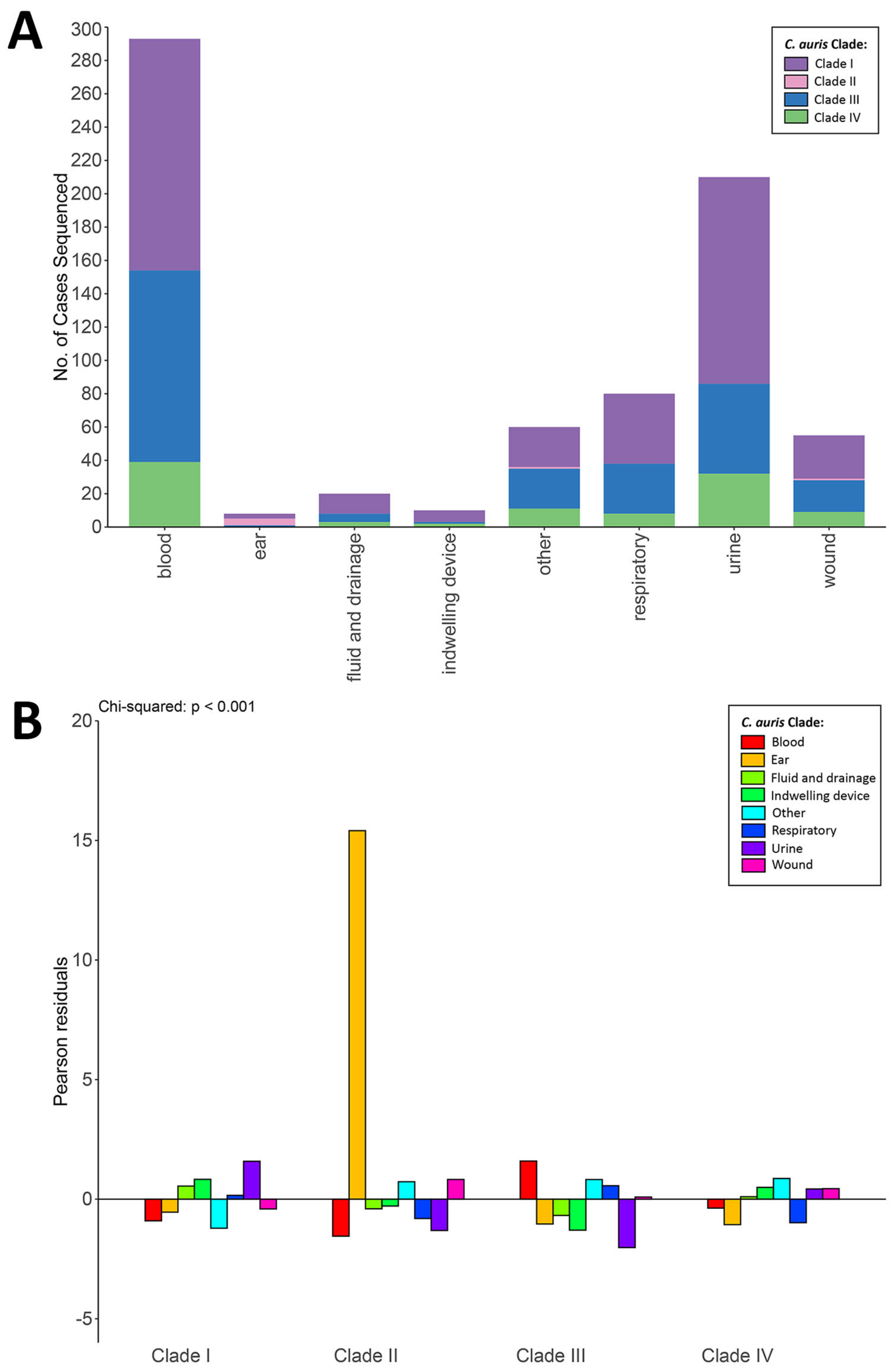

Appendix Figure 3. A) Proportion of each clade per clinical specimen type (n = 736). B) Standardized Pearson residuals from the chi-squared test indicates positive (y >0) and negative (y <0) associations between clade and various clinical specimen type categories (blood, ear, fluid and drainage, respiratory, urine, wound, indwelling device, and others).
